# CT-based deep learning auto-segmentation of high-risk clinical target volume in CT-guided cervical cancer brachytherapy: a single-center pragmatic study

**DOI:** 10.3389/fonc.2026.1936913

**Published:** 2026-09-25

**Authors:** Chen-ying Ma, Yi Fu, Lu Zhang, Lou Liu, Yu Wang, Le-cheng Jia, Wei-qi Xiong, Wei Zhang, Xiao-ting Xu, Ju-ying Zhou

**Affiliations:** 1 Department of Radiation Oncology, The First Affiliated Hospital of Soochow University, Suzhou, China; 2 Shanghai United Imaging Healthcare Co., Ltd., Shanghai, China; 3 Shenzhen United Imaging Research Institute of Innovative Medical Equipment, Shenzhen, China

**Keywords:** automated segmentation, brachytherapy, cervical cancer, CT-guided workflow, deep learning, high-risk clinical target volume

## Abstract

**Introduction:**

Computed tomography (CT)-based high-risk clinical target volume (HR-CTV) auto-segmentation has been previously investigated, but evidence remains heterogeneous across applicators, target definitions, architectures, and clinical evaluation procedures. A pragmatic within-cohort benchmark of 2D U-Net, 3D U-Net, and nnFormer was performed in a CT-only, applicator-*in-situ* workflow.

**Methods:**

CT images from 544 brachytherapy fractions in 182 patients were analyzed, including 509 fractions from 163 patients treated with tandem-and-ovoid applicators and 35 fractions from 19 patients treated with vaginal cylinders. All fractions from a patient remained in one partition (HR-CTV_c_: 325/82/102 fractions; HR-CTV_v_: 22/6/7 fractions). Only seven test fractions were available and thus the postoperative HR-CTV_v_ analysis was exploratory. Performance was assessed using Dice similarity coefficient (DSC), 95th percentile Hausdorff distance (HD95), average surface distance (ASD), and physician consensus edit categories. Task-level CT normalization parameters were estimated exclusively from the training partition and fixed for validation and testing; spatial cropping was image centered and contour independent.

**Results:**

In the intact-cervix test cohort, 3D U-Net showed the most favorable descriptive combination of overlap and surface agreement (DSC 0.848 ± 0.059; HD95 2.755 ± 1.688 mm; ASD 1.008 ± 0.622 mm). In the exploratory vaginal stump cohort, corresponding values were 0.788 ± 0.060, 4.724 ± 2.434 mm, and 2.917 ± 2.150 mm. On physician consensus review, 91/102 HR-CTV_c_ and 6/7 HR-CTV_v_ 3D U-Net contours required no or localized correction. In an ancillary 20-fraction independent-contouring analysis, physician-to-physician HR-CTV agreement was DSC 0.80 ± 0.06 and HD95 3.4 ± 1.8 mm.

**Conclusions:**

In this internally validated CT-guided cohort, 3D U-Net provided the most favorable overall performance among the evaluated architectures for intact-cervix cases. The postoperative results provide a preliminary feasibility signal, and confirmation in a larger cohort is required. These findings support further physician-supervised implementation, followed by multicenter external validation and cohort-level dosimetric assessment.

## Introduction

Cervical cancer remains a major global health burden, and image-guided adaptive brachytherapy is essential for curative treatment for locally advanced disease (1, 2). Accurate high-risk clinical target volume (HR-CTV) delineation underpins target coverage and organ-at-risk sparing (OAR) sparing (2–4). Magnetic resonance imaging (MRI) is preferred because of superior soft tissue contrast; nevertheless, computed tomography (CT)-guided planning remains common where access, scheduling, applicator compatibility, or workflow constraints limit MRI at each fraction (2–7).

Manual HR-CTV contouring on CT is time-consuming and observer dependent (3, 4). Prior studies have evaluated deep-learning segmentation in CT-based tandem-and-ovoid brachytherapy, postoperative vaginal brachytherapy, MRI-based workflows, and OAR implementation (8–17). More recent work has benchmarked convolutional neural network (CNN)-, transformer-, and state-space architectures in larger CT cohorts and incorporated physician acceptability and dosimetric endpoints (10, 16). Thus, CT-based auto-segmentation itself is no longer the unresolved question; the remaining need is transparent evaluation under deployment-relevant preprocessing, patient-separated testing, target-specific clinical review, and explicit uncertainty for small subgroups.

This study was designed as a pragmatic single-center benchmark rather than a new architecture proposal or a replacement for MRI-guided target definition. Its incremental contribution is the comparison of three representative architecture classes—slice-wise CNN, volumetric CNN, and volumetric transformer—within the same CT-only, applicator-*in-situ* workflow, covering intact-cervix tandem-and-ovoid and postoperative vaginal-cylinder target definitions. The primary objective was to compare geometric performance for intact-cervix HR-CTV (HR-CTV_c_); the postoperative vaginal stump HR-CTV (HR-CTV_v_) results were exploratory. The secondary objective was to characterize physician consensus edit burden as a clinically relevant implementation endpoint.

## Methods

### Study design and data partitioning

This retrospective single-center model development and internal validation study included 544 CT-guided brachytherapy fractions from 182 patients, including 509 fractions from 163 intact-cervix patients treated with tandem-and-ovoid applicators and 35 fractions from 19 postoperative patients treated with vaginal cylinders. All fractions from each patient were assigned to one partition only. HR-CTV_c_ fractions were divided 325/82/102 and the HR-CTV_v_ fractions were divided 22/6/7 into training/validation/testing sets, respectively. The HR-CTV_v_ test set contained seven fractions, and thus this subgroup was analyzed descriptively as exploratory.

CT was acquired with the applicator *in situ* on a Philips Brilliance Big Bore (Philips Medical Systems (Cleveland) Inc., Cleveland, OH, USA) scanner at 120 kVp and approximately 325 mAs, without intravenous contrast. Native in-plane resolution was approximately 0.66 × 0.66 mm and slice thickness was 3 mm.

### Reference contouring

The clinical reference contours followed ICRU Report 89 and IBS–GEC-ESTRO–ABS CT-based recommendations (3, 4). Contours were generated and reviewed by two senior radiation oncologists specializing in gynecologic radiotherapy, each with at least 10 years of clinical radiotherapy experience. One physician generated the initial contour, and the other performed slice-by-slice clinical review and modification as needed. Review incorporated examination findings, diagnostic MRI obtained before brachytherapy when available, external-beam records, and prior brachytherapy fractions. Disagreements were resolved by joint review before treatment approval. These CT-based consensus contours represent a pragmatic clinical reference rather than MRI-defined ground truth.

An ancillary analysis was available for 20 test fractions (16 HR-CTV_c_ and 4 HR-CTV_v_), in which the two physicians delineated HR-CTV independently while unaware of the other contour. Pairwise physician agreement was Dice similarity coefficient (DSC) 0.80 ± 0.06 and 95th percentile Hausdorff distance (HD95) 3.4 ± 1.8 mm. The corresponding 3D U-shaped convolutional neural network architecture (U-Net)-to-observer DSCs were 0.81 ± 0.06 and 0.80 ± 0.06. This geometric analysis provides an interobserver benchmark distinct from the consensus clinical-usability endpoint. The four-fraction HR-CTV_v_ component was reported descriptively.

### Models and label-independent preprocessing

The evaluated architectures were two-dimensional (2D) U-Net, three-dimensional (3D) U-Net, and nnFormer (18–20). The 2D model processed slices independently; the 3D model used volumetric convolutions; and nnFormer used volumetric self-attention. The applicator remained visible, but no applicator mask or digitization channel was supplied (9, 17). The task-specific 3D U-Net encoder–decoder configurations for HR-CTV_c_ and HR-CTV_v_ are shown in **Figures 1** and **2**, respectively.

**Figure 1:**
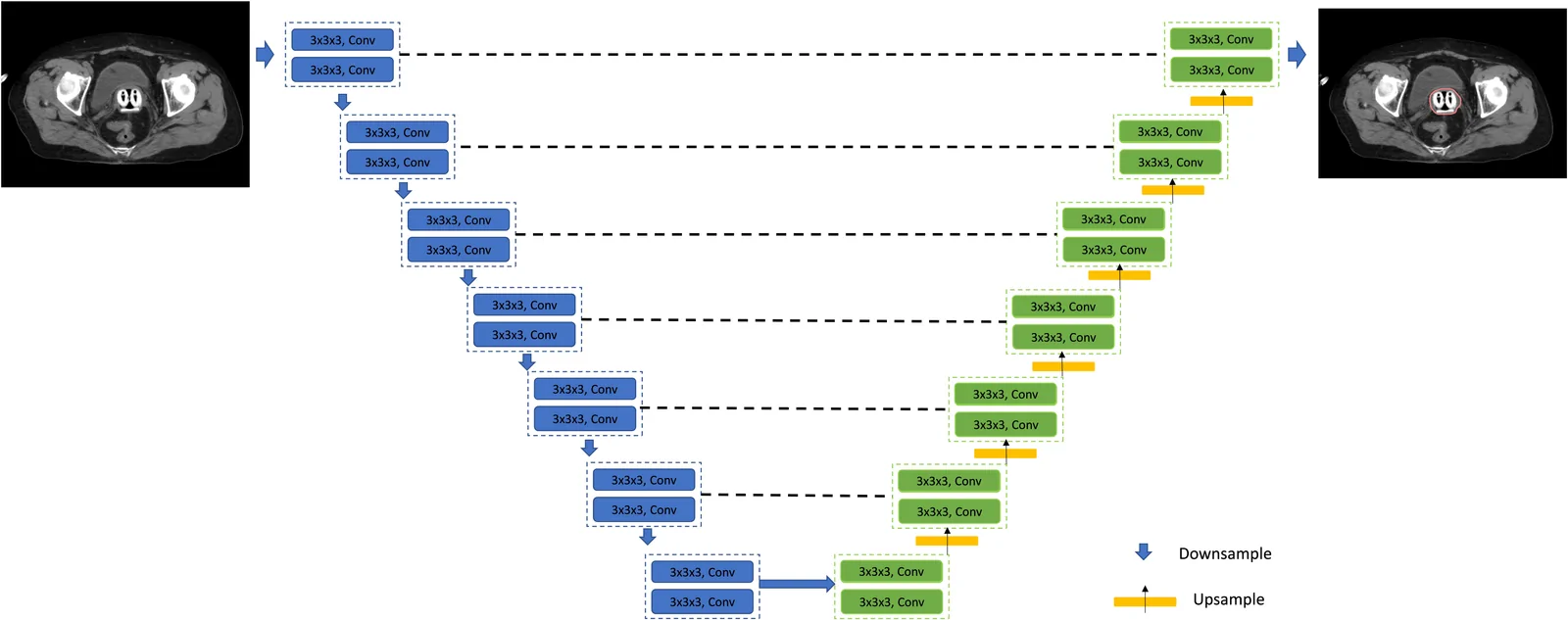
3D U-Net encoder–decoder architecture used for HR-CTV_c_ auto-segmentation in tandem-and-ovoid brachytherapy.

**Figure 2:**
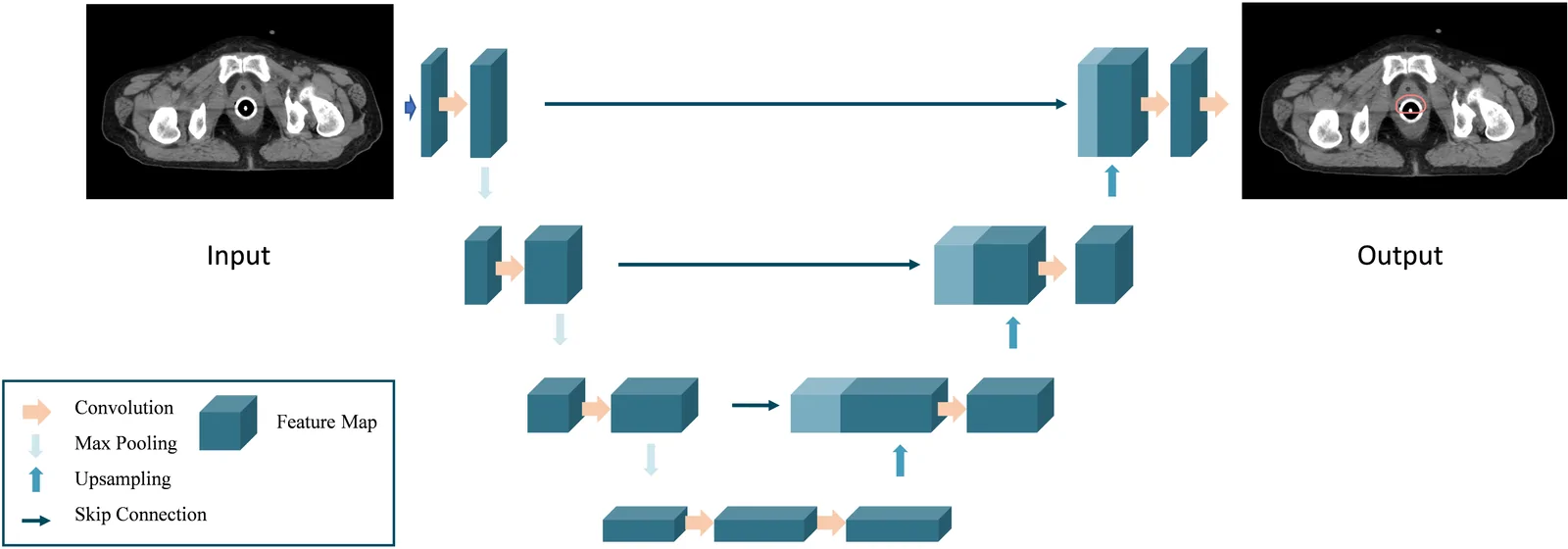
3D U-Net encoder–decoder architecture used for exploratory HR-CTV_v_ auto-segmentation in postoperative vaginal-cylinder brachytherapy.

The two clinical target types were handled as separate tasks. Each task was resampled to the median native spacing of its training cohort—3.0 × 0.6003 × 0.6003 mm^3^ for HR-CTV_c_ and 3.0 × 0.5814 × 0.5814 mm^3^ for HR-CTV_v_—using linear interpolation for CT and nearest-neighbor interpolation for masks. In accordance with the nnU-Net CT-normalization strategy (21), the 0.5th and 99.5th percentiles, mean, and standard deviation were calculated once from pooled HR-CTV foreground voxels in the training partition. These task-level parameters were then fixed and applied unchanged to all training, validation, and test images. After resampling, images were cropped or padded to 32 × 256 × 256 voxels around the image-matrix center; this operation did not use an HR-CTV contour. Validation and test labels were accessed only after inference for metric calculation. Thus, no target label was required at inference.

Training used Adam (initial learning rate 0.001), plateau-based decay, batch size 2 for volumetric models and 8 for the 2D model, a Dice-plus-cross-entropy loss, and random rotation, flipping, and intensity perturbation. Training continued for 300 epochs, and the checkpoint with the best validation DSC was selected before test evaluation (21).

### Clinical usability and dosimetric illustration

The test-set contours were reviewed by the same two senior radiation oncologists specializing in gynecologic radiotherapy using a consensus-adjudication design. For each contour, one final category was assigned. “No correction” indicated immediate clinical acceptability. “Minor correction” indicated localized boundary editing without complete redelineation. “Major correction” indicated substantial multi-slice editing, correction of a clinically important geographic miss, or complete or near-complete redelineation (8, 10, 22). Because this design generated one adjudicated category rather than paired physician-level ordinal ratings, weighted Cohen’s kappa was not applicable. Interobserver variation was instead quantified geometrically in the separate 20-fraction analysis. The category distribution is reported in **Supplementary Table S1**. The physician-supervised evaluation pathway is summarized in **Figure 3**.

**Figure 3:**
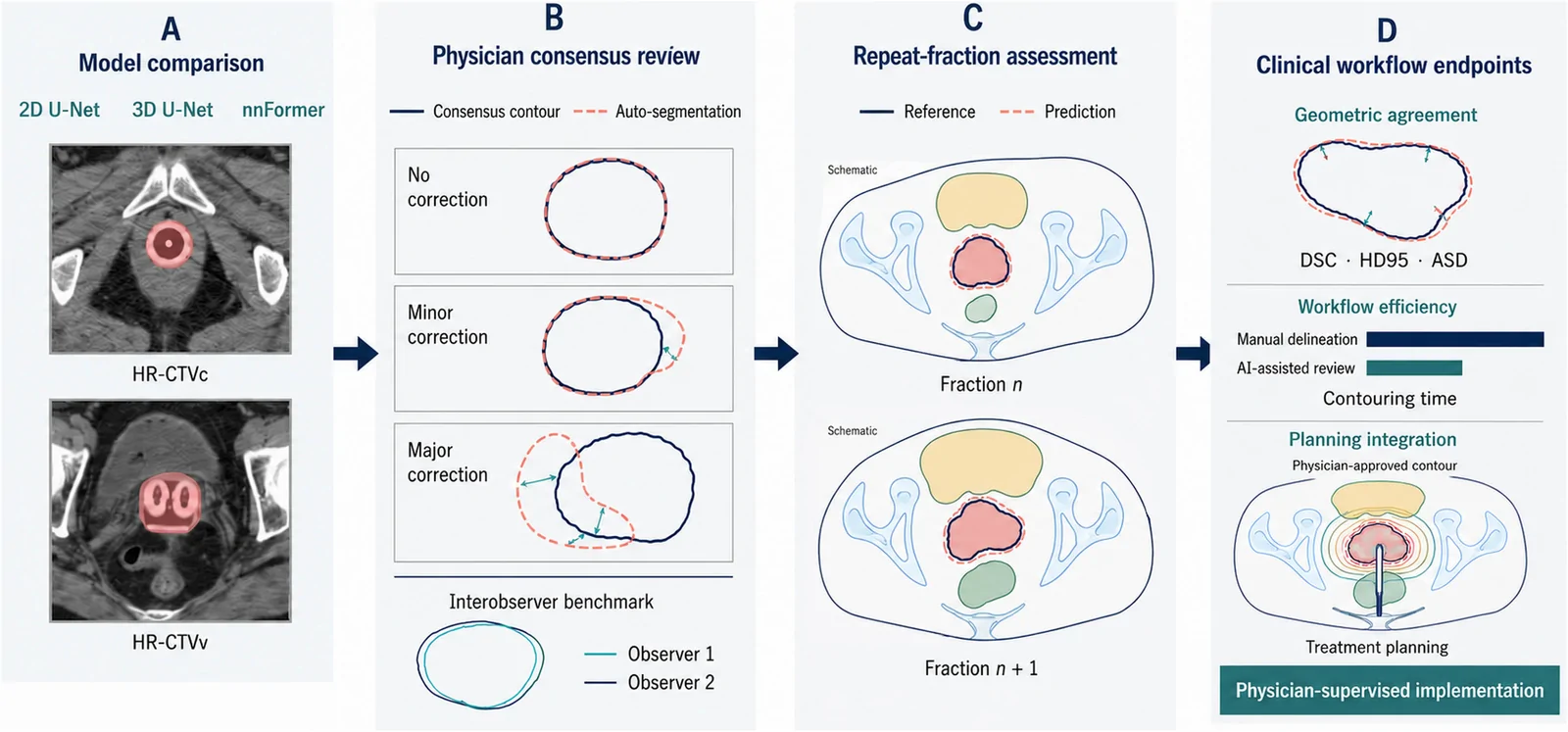
Physician-supervised evaluation and implementation pathway for CT-based auto-segmentation. **(A)** Comparison of 2D U-Net, 3D U-Net, and nnFormer for intact-cervix HR-CTV_c_ and exploratory postoperative HR-CTV_v_ tasks. **(B)** Consensus grading of contour-editing burden with a separate geometric interobserver benchmark. **(C)** Schematic repeat-fraction assessment. **(D)** Integration of geometric agreement, workflow efficiency, and physician-approved contours into treatment planning.

A predefined 20-fraction workflow evaluation included 16 HR-CTV_c_ and 4 HR-CTV_v_ fractions. Manual time covered full HR-CTV and OAR delineation. The assisted pathway combined treatment-planning-system OAR auto-contouring, 3D U-Net HR-CTV inference, and physician review and correction of the generated structures. Total assisted time therefore represented the complete clinical contouring workflow rather than inference alone. **Supplementary Table S2** reproduces the archived aggregate analysis using mean ± standard deviation (SD) and median (IQR). As this was a descriptive implementation endpoint, no *post hoc* case-level inferential analysis was introduced.

Dose-volume histograms (DVHs) from one tandem-and-ovoid case and one vaginal-cylinder case were selected to demonstrate how contour variation may propagate to treatment-planning metrics. This component was qualitative by design and was not an equivalence or noninferiority test.

### Statistical analysis

Segmentation performance was evaluated using DSC, HD95, and average surface distance (ASD) (23–25). Test-set results are reported as mean ± SD, consistent with the locked aggregate analysis. The large nnFormer HR-CTV_c_ HD95 dispersion was interpreted as an outlier-sensitive distribution in accordance with current validation guidance (26, 27). Because the retained output set comprised aggregate rather than case-level metrics, median, IQR, and empty-prediction frequency were not estimated retrospectively. **Table 1** reports the original fraction-level pairwise comparisons. Given within-patient clustering of repeated fractions, these *P* values are interpreted as exploratory descriptive evidence; architecture selection was based on the combined geometric and clinical-review findings rather than significance testing alone.

**Table 1:** Exploratory fraction-level pairwise comparison of segmentation metrics among the three evaluated models.

Model comparison	HR-CTV_c_			HR-CTV_v_		
	DSCs	95 HD	ASD	DSCs	95 HD	ASD
2D U-Net *vs.* 3D U-Net	<0.01	<0.01	<0.01	0.238	0.193	0.235
2D U-Net *vs.* nnFormer	0.035	0.159	0.239	0.013	0.154	0.084
3D U-Net *vs.* nnFormer	<0.01	0.133	0.077	0.032	0.352	0.306

DSC, Dice similarity coefficient; HD95, 95th percentile Hausdorff distance; ASD, average surface distance; HR-CTV_c_, cervical high-risk clinical target volume; HR-CTV_v_, vaginal stump high-risk clinical target volume. Pairwise comparisons were performed at the fraction level and should be interpreted as exploratory. Because multiple fractions from the same patient were included, the assumption of independent observations was not fully met; therefore, *P* values are presented for descriptive context only and should not be interpreted as confirmatory evidence of patient-level superiority.

## Results

### Cohort characteristics

The intact-cervix cohort included International Federation of Gynecology and Obstetrics stages IB3–IVA. No interstitial or hybrid-needle cases were included. The median number of fractions per patient was 3 in the intact-cervix group and 2 in the vaginal-cylinder group (**Table 2**).

**Table 2:** Patient characteristics.

Characteristic	HR-CTV_c_ / Intracavitary T&O (n = 163)	HR-CTV_v_ / Vaginal cylinder (n = 19)	Overall (n = 182)
Age, years	54 (48–61)	58 (52–65)	55 (49–62)
FIGO stage, n (%)			
IB_3_	22 (13.5%)	0 (0%)	22 (12.1%)
IIA–IIB	68 (41.7%)	3 (15.8%)	71 (39.0%)
IIIA–IIIC_2_	63 (38.7%)	2 (10.5%)	65 (35.7%)
IVA	10 (6.1%)	0 (0%)	10 (5.5%)
Postoperative / recurrent vaginal stump	—	14 (73.7%)	14 (7.7%)
HR-CTV volume at first BT (cm³)	32.6 (22.8–45.3)	18.5 (11.6–25.2)	30.7 (20.9–43.7)
Extent of disease at BT, n (%)			
Confined to cervix	51 (31.3%)	—	51 (28.0%)
Mid/upper uterine involvement	23 (14.1%)	—	23 (12.6%)
Parametrial involvement-proximal	47 (28.8%)	—	47 (25.8%)
Parametrial involvement distal / pelvic wall	28 (17.2%)	—	28 (15.4%)
Vaginal extension	9 (5.5%)	5 (26.3%)	14 (7.7%)
Bladder/rectal invasion	5 (3.1%)	0 (0%)	5 (2.7%)
BT technique			
Type III tandem-and-ovoid intracavitary	163 (100%)	—	163 (89.6%)
Vaginal cylinder intracavitary	—	19 (100%)	19 (10.4%)
Interstitial / hybrid needles	0 (0%)	0 (0%)	0 (0%)
Fractions per patient, median [IQR]	3 (3–4)	2 (2–3)	3 (2–4)

FIGO, International Federation of Gynecology and Obstetrics; HR-CTV, high-risk clinical target volume; BT, brachytherapy; IQR, interquartile range.

### Segmentation performance

Preprocessing at validation and test time used fixed training-derived CT statistics and contour-independent center cropping, so no target label was required for inference. The resulting test-set performance is summarized **Table 3**. For HR-CTV_c_, 3D U-Net showed the most favorable descriptive combination of DSC, HD95, and ASD.

**Table 3:** Automated segmentation of HR-CTV_c_ and HR-CTV_v_ by 3D U-Net, 2D U-Net, and nnFormer.

Target volume	Network	DSC (mean ± SD)	HD95 (mm, mean ± SD)	ASD (mm, mean ± SD)
HR-CTV_c_	3D U-Net	0.848 ± 0.059	2.755 ± 1.688	1.008 ± 0.622
	2D U-Net	0.817 ± 0.061	3.588 ± 2.196	1.580 ± 1.744
	nnFormer	0.806 ± 0.061	10.360 ± 68.329	2.145 ± 7.981
HR-CTV_v_	3D U-Net	0.788 ± 0.060	4.724 ± 2.434	2.917 ± 2.150
	2D U-Net	0.766 ± 0.093	5.952 ± 3.117	2.176 ± 0.890
	nnFormer	0.706 ± 0.091	5.034 ± 1.523	2.462 ± 0.787

DSC, Dice similarity coefficient; HD95, 95th percentile Hausdorff distance; ASD, average surface distance; HR-CTV_c_, cervical high-risk clinical target volume; HR-CTV_v_, vaginal stump high-risk clinical target volume. The large dispersion of the nnFormer HR-CTV_c_ surface-distance results indicates a markedly skewed, outlier-sensitive distribution. The archived analysis reported aggregate endpoints; no unverified median, IQR, or empty-prediction frequency was estimated retrospectively. HR-CTV_v_ estimates are exploratory because the test set contained seven fractions.

**Table 1** presents the fraction-level pairwise comparisons as exploratory descriptive evidence. Because repeated fractions were clustered within patients, these *P* values were not interpreted as confirmatory patient-level tests.

The nnFormer HR-CTV_c_ HD95 was 10.360 ± 68.329 mm. The disproportionately large SD indicates a markedly right-skewed, outlier-sensitive distribution, making the mean less representative of typical performance (26, 27). The archived output set contained aggregate rather than case-level HD95 values and empty-mask indicators; accordingly, no unverified median, IQR, or empty-prediction count was introduced. Model performance was interpreted across DSC, HD95, ASD, and physician consensus review rather than from this metric in isolation.

For HR-CTV_v_, the seven test fractions provide preliminary descriptive estimates of technical feasibility. Confirmation of the numerical architecture ranking and its transportability requires a larger postoperative cohort. Representative multi-planar outputs for both target types are shown in **Figure 4**.

**Figure 4:**
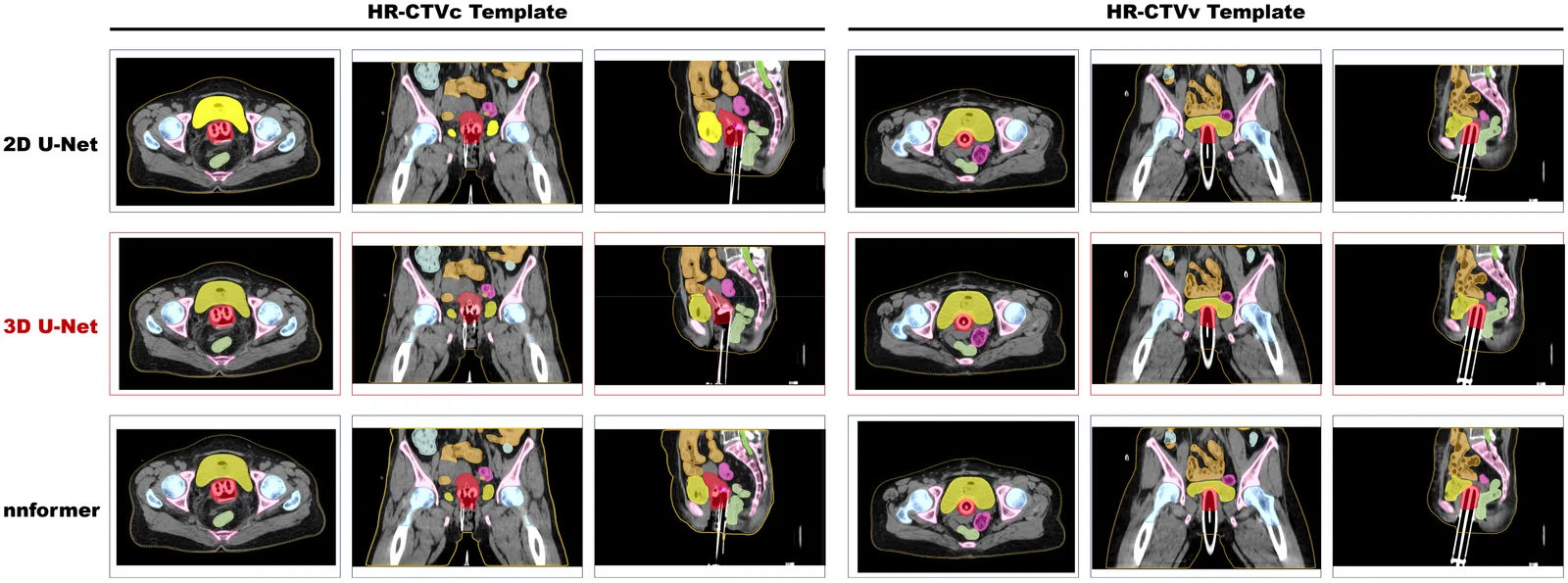
Representative multi-planar comparison of predictions from 2D U-Net, 3D U-Net, and nnFormer for intact-cervix HR-CTV_c_ and exploratory postoperative HR-CTV_v_ cases. (Red, HR-CTV; yellow, bladder; green, rectum; magenta, sigmoid colon; beige, small intestine; light pink, pelvis; blue, femoral head; HR-CTV_c_ was used for target volume delineation in radical cervical cancer brachytherapy; HR-CTV_v_ was used for target volume delineation in vaginal stump brachytherapy).

### Physician consensus review

On consensus review, 3D U-Net required no or minor correction in 91/102 HR-CTV_c_ fractions (89.2%) and 6/7 HR-CTV_v_ fractions (85.7%). The HR-CTV_v_ estimate is exploratory because it was based on seven test fractions. The consensus-adjudication design generated one final category per contour, so weighted kappa was not applicable. The complete distribution for all three models is provided in **Supplementary Table S1**.

### Descriptive workflow timing

In the predefined 20-fraction workflow evaluation, full manual HR-CTV and OAR delineation required 24.3 ± 2.9 min per fraction (median, 24.0 min; IQR, 22.1–26.5 min). The 3D U-Net inference time was 5.4 ± 0.8 s, and the total assisted contouring workflow required 3.2 ± 0.5 min (median, 3.1 min; IQR, 2.8–3.5 min). The corresponding aggregate absolute reduction was 21.1 ± 2.8 min and the relative reduction was 86.8% ± 3.4% (**Supplementary Table S2**). These results are reported as a descriptive implementation endpoint, consistent with the archived analysis plan.

### Illustrative DVH cases

In the two selected examples, manual- and auto-contour-based curves appeared similar (**Figure 5**). These observations are presented as qualitative planning illustrations; cohort-level equivalence, safety, and noninferiority were outside the design of this component.

**Figure 5:**
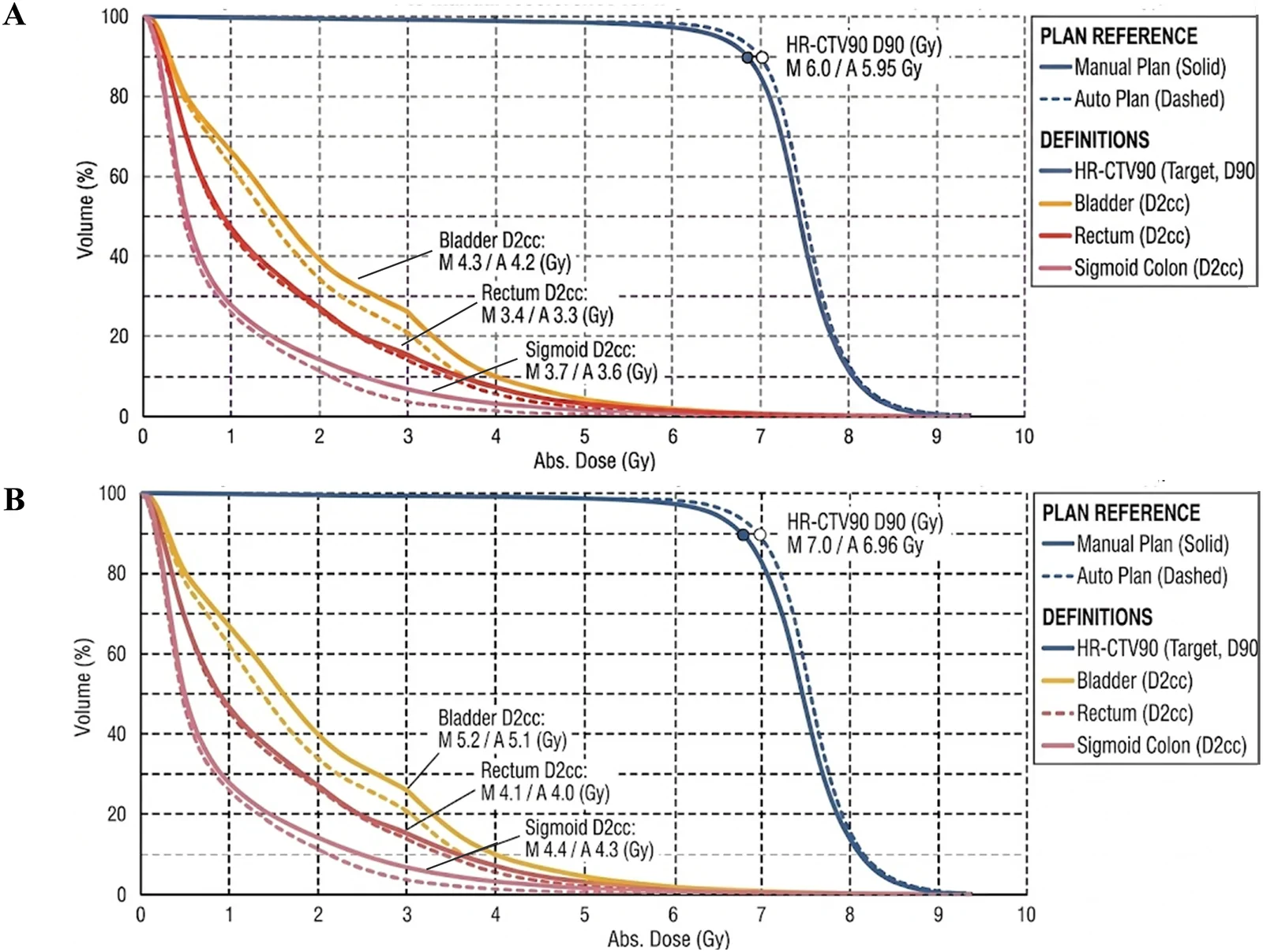
Two representative DVH comparisons of manual- and auto-contour-based plans. **(A)** tandem-and-ovoid case and **(B)** vaginal-cylinder case. Solid lines indicate manual-contour-based plans and dashed lines auto-contour-based plans. HR-CTV D90 and OAR D_2cc_ use standard brachytherapy notation. These selected examples are descriptive and do not constitute a cohort-level dosimetric equivalence analysis. (HR-CTV_c_ was used for target volume delineation in radical cervical cancer brachytherapy; HR-CTV_v_ was used for target volume delineation in vaginal stump brachytherapy).

## Discussion

The central finding is clinically pragmatic: within a CT-only, applicator-*in-situ* cohort, the volumetric 3D U-Net produced the most favorable overall balance of overlap, boundary agreement, and consensus edit burden for intact-cervix cases. This result prioritizes 3D U-Net for physician-supervised implementation and external validation in comparable brachytherapy workflows.

Prior CT-guided brachytherapy studies addressed applicator-aware segmentation, postoperative target delineation, and dedicated tandem-and-ovoid HR-CTV models (9–11, 16, 17). Wang et al. reported a postoperative HR-CTV DSC of 0.87 and combined geometric, physician, and dosimetric assessment (10). Kraus et al. prospectively evaluated OAR auto-contouring in CT-based gynecologic brachytherapy and emphasized the distinction between technical inference and clinically verified workflow efficiency (22). In a 2025 five-model benchmark of 694 CT scans, Zang et al. reported an HR-CTV DSC of 0.862 and HD95 of 3.638 mm, together with physician acceptability and cohort-level dosimetry (16). Related cervical cancer segmentation studies in planning CT, MRI, and dual-modality workflows have broadened technical and clinical validation across target and OAR settings (12–15, 28–38). The present study therefore provides a patient-separated, within-cohort comparison across three architecture classes and two target definitions rather than a first demonstration of CT auto-segmentation.

CT-guided workflow remains clinically relevant because radiotherapy and imaging resources are unequally distributed (5, 6), while applicator-*in-situ* MRI may be constrained by access, scheduling, and compatibility (7). Hybrid MRI/CT strategies can reduce dependence on MRI at every fraction (39). Nevertheless, CT-based automation still requires explicit recognition of the limitations of CT-defined HR-CTV.

The apparent advantage of 3D U-Net is technically plausible because HR-CTV definition depends on volumetric relationships among residual disease, cervix or vaginal cuff, uterus, parametria, and applicator geometry (18–21). Slice-wise models cannot directly enforce inter-slice coherence, whereas transformer-based models may be more sensitive to cohort size and optimization (20, 21, 40, 41). Semi-supervised MRI-based OAR segmentation has expanded potential training strategies (42), but it does not remove the target-definition limitations of CT-only HR-CTV segmentation. The very large nnFormer HR-CTV_c_ HD95 dispersion illustrates why a single mean can obscure clinically important failure behavior. Recent validation guidance recommends matching metrics to the intended clinical use, examining distributions and outliers, and combining geometric results with structured expert review rather than relying on one aggregate measure (26, 27, 43).

The ancillary physician-to-physician analysis provides a clinically relevant benchmark for contour variability in this CT-based workflow and complements the consensus edit categories. In a 2026 multicenter study, Peng et al. reported a baseline interobserver DSC of 0.88 and HD95 of 3.2 mm, with further improvement after limited clinician prompting (44). Differences from the present DSC of 0.80 and HD95 of 3.4 mm are plausible given the CT-only target definition, cohort composition, and delineation procedure. Together, these findings support supervised use and explicit reporting of observer variability.

The predefined 20-fraction workflow evaluation demonstrated a substantial reduction in contouring time within the implemented clinical pathway. This endpoint represents an aggregate implementation signal rather than a prospective time-motion comparison because the assisted workflow combined OAR automation, HR-CTV inference, and physician review. A prospective study with case-level paired timing and standardized workflow definitions would establish the precision and transportability of this efficiency estimate.

The seven postoperative test fractions provide a preliminary feasibility signal for vaginal stump auto-segmentation. Their limited anatomical coverage and statistical precision do not support definitive architecture ranking, but the observed performance provides a clinically relevant basis for expanding postoperative cohorts and prospective workflow evaluation (10, 22).

The two DVH examples illustrate how geometric contour differences propagate to treatment-planning metrics; they were not designed to estimate population-level dosimetric impact. The appropriate confirmatory assessment is locked-protocol replanning of the complete test cohort with paired HR-CTV D90 and OAR D_2cc_ differences, confidence intervals, and prespecified clinically meaningful thresholds (10, 16, 22, 43).

Several design features define the scope of inference. Reference contours were CT-based clinical consensus contours rather than MRI-defined targets, and internal validation was performed within a single acquisition and contouring environment with repeated fractions and a small postoperative subgroup (3, 4). The primary intact-cervix findings therefore establish performance within the intended institutional workflow, while multicenter testing is required to assess transportability. Inference itself was label independent, although the training-derived intensity fingerprint remains institution and task specific (21). Clinical usability was evaluated by consensus adjudication, for which weighted kappa is not applicable. The archived nnFormer and timing outputs were aggregate, so *post hoc* case-level distributional and paired timing analyses were not introduced. These boundaries support physician-supervised implementation and define the next validation steps without altering the reported internal test results.

## Conclusions

In this internally validated CT-guided brachytherapy cohort, 3D U-Net showed the most favorable overall performance among the evaluated architectures for intact-cervix cases and is the leading candidate for physician-supervised implementation in this workflow. The postoperative vaginal stump results provide a preliminary feasibility signal that requires confirmation in a larger cohort. Multicenter external validation, interobserver benchmarking, and cohort-level dosimetric assessment are the appropriate next steps. MRI-guided target definition remains preferred where available.

## Data Availability

The original contributions presented in the study are included in the article/**Supplementary Material**. Further inquiries can be directed to the corresponding author.
