# Supplementary material for: CT-based deep learning auto-segmentation of high-risk clinical target volume in CT-guided cervical cancer brachytherapy: a single-center pragmatic study
Source: Front Oncol. 2026 Sep 25;16:1936913. doi: 10.3389/fonc.2026.1936913 (PMC13649455; doi:10.3389/fonc.2026.1936913)
Supplement: Supplementary file 1 [file Table1.doc]

**Table S1. Qualitative physician-review categories for HR-CTV auto-segmentation in the test cohort**

| Target | Model | No edit, n (%) | Minor edit, n (%) | Major edit, n (%) | No or minor edit, n (%) |
| --- | --- | --- | --- | --- | --- |
| HR-CTV_c_ (n = 102) | 3D-Unet | 38 (37.3%) | 53 (52.0%) | 11 (10.8%) | 91 (89.2%) |
| HR-CTV_c_ (n = 102) | 2D-Unet | 20 (19.6%) | 52 (51.0%) | 30 (29.4%) | 72 (70.6%) |
| HR-CTV_c_ (n = 102) | nnFormer | 15 (14.7%) | 49 (48.0%) | 38 (37.3%) | 64 (62.7%) |
| HR-CTV_v_ (n = 7) | 3D-Unet | 1 (14.3%) | 5 (71.4%) | 1 (14.3%) | 6 (85.7%) |
| HR-CTV_v_ (n = 7) | 2D-Unet | 1 (14.3%) | 3 (42.9%) | 3 (42.9%) | 4 (57.1%) |
| HR-CTV_v_ (n = 7) | nnFormer | 0 (0.0%) | 3 (42.9%) | 4 (57.1%) | 3 (42.9%) |

Note: HR-CTV_c_, Cervical high-risk clinical target volume; HR-CTV_v_, Vaginal stump high-risk clinical target volume. Clinical usability was evaluated using a consensus-adjudication design, with one final category assigned to each model-generated contour. “No correction” denoted immediate clinical acceptability; “minor correction” denoted localized boundary editing without complete redelineation; and “major correction” denoted substantial multi-slice editing, correction of a clinically important geographic miss, or complete or near-complete redelineation. Because the endpoint did not generate paired physician-level ordinal ratings, weighted Cohen’s kappa was not applicable.

**Table S2.** Descriptive workflow timing summary for manual and 3D U-Net-assisted contouring

| Workflow component | Sample | Mean ± SD | Median (IQR) |
| --- | --- | --- | --- |
| Manual contouring (HR-CTV + OARs) | 20 fractions | 24.3 ± 2.9 min | 24.0 (22.1–26.5) min |
| 3D-Unet inference time | 20 fractions | 5.4 ± 0.8 s | 5.2 (4.9–6.0) s |
| Physician review/correction after auto-segmentation | 20 fractions | 3.1 ± 0.5 min | 3.0 (2.7–3.4) min |
| Total assisted workflow time | 20 fractions | 3.2 ± 0.5 min | 3.1 (2.8–3.5) min |
| Absolute time saved | 20 fractions | 21.1 ± 2.8 min | 20.9 (19.1–23.3) min |
| Relative time reduction | 20 fractions | 86.8% ± 3.4% | 87.0% (84.2%–89.1%) |

Note: HR-CTV, high-risk clinical target volume; OAR, organ at risk; SD, standard deviation; IQR, interquartile range. Manual contouring time included full HR-CTV and OAR delineation. The assisted pathway combined treatment-planning-system OAR auto-contouring, 3D U-Net HR-CTV inference, and physician review and correction of the generated structures. Total assisted workflow time therefore includes inference and physician review/correction. Values reproduce the predefined archived aggregate analysis and are presented as descriptive implementation evidence; no post hoc inferential statistics were introduced.
